# Similarity of Individuals and Social Organisms

**Published:** 1876-09

**Authors:** Herbert Spencer


### *SIMILARITY OF THE INDIVIDUAL AND SOCIAL ORGANISMS.*

Conversely, in both cases, if not brought to a close by violence, the life of the aggregate greatly exceeds in duration the lives of its units. The minute living elements composing a developed animal severally evolve, play their parts, decay, and are replaced, while the animal as a whole continues. In the deep layer of the skin, cells are formed by fission, which, as they enlarge, are thrust outward, and, becoming flattened to form the epidermis, eventually exfoliate, while the younger ones beneath take their places. Liver-cells, growing by imbibition of matters from which they separate the bile, presently die, and their vacant seats are occupied by another generation. Even bone, though so dense and seemingly inert, is permeated by blood-vessels carrying materials to replace old components by new ones. And the replacement, rapid in some tissues and in others slow, goes on at such rate that, during the continued existence of the entire body, each portion of it has been many times over produced and destroyed. Thus it is also with a society and its units. Integrity of the whole and of each large division is perennially maintained, notwithstanding the deaths of component citzens. The fabric of living persons, which, in a manufacturing town, produces some commodity for national use, remains after a century as large a fabric, though all the masters and workers who a century ago composed it have long since disappeared. Even with the minor parts of this industrial structure the like holds. A firm that dates from past generations, still carrying on business in the name of its founder, has had all its members and employés changed one by one, perhaps several times over, while the firm has continued to occupy the same place and to maintain like relations to buyers and sellers. Throughout we find this. Governing bodies, general and local, ecclesiastical corporations, armies, institutions of all orders down to guilds, clubs, philanthropic associations, etc., show us a continuity of life exceeding that of the person constituting them. Nay, more. As part of the same law, we see that the existence of the society at large exceeds in duration that of some of these compound parts. Private unions, local public bodies, secondary national institutions, towns carrying on special industries, may decay, while the nation maintaining its integrity, evolves in mass and structure. —Herbert Spencer, *in Popular Science Monthly for May.*

